# Factors influencing identification of and response to intimate partner violence: a survey of physicians and nurses

**DOI:** 10.1186/1471-2458-7-12

**Published:** 2007-01-24

**Authors:** Iris Gutmanis, Charlene Beynon, Leslie Tutty, C Nadine Wathen, Harriet L MacMillan

**Affiliations:** 1 Department of Epidemiology and Biostatistics, Schulich School of Medicine & Dentistry, The University of Western Ontario, London, Canada; 2 Research, Education, Evaluation, and Development Services, Middlesex-London Health Unit, and School of Nursing, Faculty of Health Sciences, The University of Western Ontario, London, Canada; 3 Faculty of Social Work, University of Calgary, Calgary, Canada; 4 Faculty of Information Studies, University of Toronto, Toronto, Canada; 5 Departments of Psychiatry and Behavioural Neurosciences, and of Pediatrics, McMaster University, Hamilton, Canada

## Abstract

**Background:**

Intimate partner violence against women (IPV) has been identified as a serious public health problem. Although the health care system is an important site for identification and intervention, there have been challenges in determining how health care professionals can best address this issue in practice. We surveyed nurses and physicians in 2004 regarding their attitudes and behaviours with respect to IPV, including whether they routinely inquire about IPV, as well as potentially relevant barriers, facilitators, experiential, and practice-related factors.

**Methods:**

A modified Dillman Tailored Design approach was used to survey 1000 nurses and 1000 physicians by mail in Ontario, Canada. Respondents were randomly selected from professional directories and represented practice areas pre-identified from the literature as those most likely to care for women at the point of initial IPV disclosure: family practice, obstetrics and gynecology, emergency care, maternal/newborn care, and public health. The survey instrument had a case-based scenario followed by 43 questions asking about behaviours and resources specific to woman abuse.

**Results:**

In total, 931 questionnaires were returned; 597 by nurses (59.7% response rate) and 328 by physicians (32.8% response rate). Overall, 32% of nurses and 42% of physicians reported routinely initiating the topic of IPV in practice. Principal components analysis identified eight constructs related to whether routine inquiry was conducted: preparedness, self-confidence, professional supports, abuse inquiry, practitioner consequences of asking, comfort following disclosure, practitioner lack of control, and practice pressures. Each construct was analyzed according to a number of related issues, including clinician training and experience with woman abuse, area of practice, and type of health care provider. Preparedness emerged as a key construct related to whether respondents routinely initiated the topic of IPV.

**Conclusion:**

The present study provides new insight into the factors that facilitate and impede clinicians' decisions to address the issue of IPV with their female patients. Inadequate preparation, both educational and experiential, emerged as a key barrier to routine inquiry, as did the importance of the "real world" pressures associated with the daily context of primary care practice.

## Background

Intimate partner violence against women (IPV) has been identified as a major public health problem [1] with serious health consequences for women and children [2-5] and significant societal impact, including high financial costs [6]. In Canada, and consistent with rates in the United States, almost 1 in 10 women are physically abused by an intimate partner in any given year, and as many as half of Canadian women report some form of physical or mental abuse over the course of their lifetime [7-9].

In health care settings, the best approach to identifying women exposed to violence remains unclear, with several systematic reviews finding insufficient evidence regarding the effectiveness of universal IPV screening in improving outcomes for women, primarily due to lack of evaluation of the interventions to which women are referred [10-12].

In the absence of evidence regarding universal screening, one approach to the identification of woman abuse in health care settings, which is supported by several national organizations [13,14], is routine inquiry when signs or symptoms of abuse are present. This "diagnostic" or "case finding" approach requires awareness by the clinician of factors associated with abuse, including physical injuries, mental health symptoms, and relationship issues shown to be related to recent or current abuse [15,16].

Whether through universal screening or case finding, a number of studies have shown that rates of routine inquiry about woman abuse by health care providers (HCPs) are generally quite low – in the range of 5–10% in primary care settings [17-19], and anywhere from 5% [19] to 25% in emergency care settings [20]. Women presenting to emergency departments with injuries consistent with IPV are asked about violence more often, but the largest study [18] found an abuse inquiry rate of just under 80% in this group.

A number of studies have examined the knowledge, attitudes, and beliefs of physicians [e.g., [21-24]] and/or nurses [e.g., [25,26]] and other health care providers [e.g., [27]] to identification of IPV. While no recent systematic review exists, the common themes that emerge from these and other studies include: gaps in provider knowledge and lack of education regarding IPV; the perception of a lack of patient compliance (i.e., patient does not disclose); lack of effective interventions and perceived system support, especially time; provider self-efficacy, including feelings of powerlessness and loss of control; safety and confidentiality concerns; fear of offending; affective barriers (e.g., lack of comfort, interest, and sympathy); poor interviewing or communication skills; providers' personal experience with abuse; fears about legal involvement; and provider age and years in practice.

The primary objectives of the present study were to identify specific barriers and facilitators to routine inquiry regarding IPV and to evaluate whether these barriers and facilitators are a function of provider type, demographic, experiential or practice-related factors among randomly selected samples of nurses and physicians most likely to care for women at the point of initial IPV disclosure.

## Methods

### Study sample

As recommended by the College of Family Physicians of Canada, the mailing list for physicians practicing in the province of Ontario, Canada, was obtained from Scott's Directories, a company that produces an annual Canadian medical and physician directory. This list included general practitioners as well as specialists employed in family practice, emergency medicine, obstetrics and gynecology, and public health. A random sample weighted by specialty/primary interest was generated using SPSS v. 11 (Chicago, Illinois). The College of Nurses of Ontario provided the mailing list for Registered Nurses employed in Ontario in family practice/physician offices, emergency care, maternal/newborn, and public health. A random sample of nurses from these practice areas was generated using SPSS. A weighted sample for nurses was not possible as the proportion in each practice area was not available. The survey period was from March to June 2004.

### Sample size calculations

Sample size calculations indicated that 994 respondents would be required assuming the following: a maximum acceptable difference between the population proportion and the sample estimate of 5%; a 95% confidence interval; two-tailed tests of significance; and a 35% response rate; which is common for surveys of clinicians [e.g., [19,28]]. As the planned analysis was profession-specific, 1000 nurses and 1000 physicians were invited to participate.

### Questionnaire design

Questionnaire items were generated through a review of the literature and an examination of instruments in the literature, particularly work by Dickson and Tutty [29] and Moore et al. [30]. Items were also generated through conversations with experts in the field. The draft 43-item questionnaire was then reviewed by a panel of experts in family violence, violence against women, nursing, and family practice who agreed that key barriers and facilitators had been identified. A copy of the questionnaire is available from the authors on request.

The questionnaire began with a practice scenario regarding a patient named "Carol" (see Additional file 1). Respondents were then asked to respond to 43 statements created to reflect either a barrier or a facilitator to their current practices specific to routine inquiry – termed "screening" in the survey questions – using either their own experience or the provided scenario. For each statement, respondents were asked to select one of four possible responses: "strongly agree", "agree", "disagree", and "strongly disagree". The 43 items were followed by a series of questions regarding respondent demographic characteristics, education and training, and experience (clinical and personal) with woman abuse. Respondents were then asked two open-ended questions regarding barriers and facilitators to screening ("What do you experience as barriers to screening for woman abuse?" and "What has helped or would help make screening for woman abuse easier for you?").

### Survey methods

Consistent with a modified Tailored Design Method [31], one week before the study questionnaire was distributed potential respondents were sent a pre-notice letter informing them of the forthcoming survey. Respondents were then sent the study questionnaire, a personalized Letter of Information including contact information if there were questions or concerns, and a two-dollar gift certificate to an Ontario-wide coffee shop. Three weeks later, all potential respondents were sent a reminder letter and replacement questionnaire.

All of the elements of consent were documented for the study participants in the Letter of Information. By returning a completed study questionnaire, it was assumed that the respondent had consented to the study. Thus, informed consent was implied. The study protocol was approved by The University of Western Ontario's Research Ethics Board.

### Data analysis

The demographic characteristics of the study sample were calculated and the proportion of responding physicians by practice area was compared to that in Scott's Directory. Then, the proportion that endorsed each response option to each question was determined, score distributions were examined, and profession-specific comparisons were made.

Using preliminary results of common categories from the two open-ended questions regarding barriers and facilitators to IPV screening, the 43 questionnaire items were grouped into possible constructs by two of the study investigators (IG and CB). For each potential construct, an item analysis was conducted followed by a Principal Components Analysis (PCA). Prior to item analysis, some items were recoded to ensure that higher scores reflected more "positive" responses. Item-item and item-total correlations were determined as well as the kurtosis and skewness associated with each construct scale. Chronbach's alpha was used to assess scale internal consistency and PCA with varimax rotation was conducted to examine the structure of each proposed scale. If eigenvalues exceeded one and the scree plot suggested more than one factor, the proposed scale items were reviewed and reallocated to new scales based on item-item and item-total correlations, and then evaluated.

This was followed by an examination of scale construct validity. It was postulated that for all of the constructs, scale scores would increase with experience with abuse disclosure. Respondents were asked, "As part of your practice, have any women disclosed abuse directly to you?", and if yes, "Approximately how many women have disclosed abuse to you in the past year?". For the latter question, respondents were offered five options (0, 1–19, 20–49, 50–99 and 100 or more). As very few respondents had experienced more than 20 abuse disclosures in the last year, the latter three response categories were collapsed to one category, 20 or more disclosures. For each construct, an analysis of variance (ANOVA) was used to compare the mean scores associated with each abuse disclosure category. As group variances were not homogeneous, a Dunnett's t-test was used to compare means. In addition, to compensate for multiple testing, differences were considered significant at the p < 0.01 level.

Mean scale scores were also examined by training and by experience. Four training and experience categories were created using the responses to two questions ("Have you ever received any formal training regarding Violence Against Women?" and "As part of your practice, have any women disclosed abuse directly to you?"). Again, for each construct, an ANOVA was used to compare the mean scores associated with each level of training and experience (Dunnetts's t-test, p < 0.01).

To facilitate comparisons between scale score changes for specific behaviours thought to be associated with all of the constructs, scores for each of the scales were summed and averaged, resulting in a score ranging from one to four for each construct.

Finally, exploratory linear regression models were built to identify demographic and experiential factors associated with preparedness. Independent variables included: four demographic variables – profession (nurse vs. physician), practice setting (rural vs. urban, as identified by the respondent), sex (male vs. female), and years in current practice location (less than 10 years vs. 10 or more years); two dummy variables associated with frequency of abuse disclosure in the last year (professional experience); personal experience (no personal experience vs. respondent, friend, or relative has had experience with abuse); and two additional experiential variables. At the end of the questionnaire, respondents were asked about their experiences with violence against women. Specifically, respondents were asked, "Have you ever had to call the police due to a disclosure of abuse?" and "Have you ever had to call the Children's Aid Society (CAS) subsequent to a disclosure of woman abuse?". These questions did not stipulate if calls to either the police or the CAS were related to clinical practice or to personal experience. Forward, backward, and stepwise regression models were generated and compared. Final model residual diagnostics were performed. Most analyses were conducted with either SPSS v.10, v. 11 or v. 14; PEPI v. 4.0 was used to calculate initial Z-tests.

## Results

### Participants

In total, 931 individuals returned completed questionnaires; 597 identified themselves as nurses (59.7% response rate) and 328 indicated that they were physicians (32.8% response rate). Six respondents did not identify their discipline. As seen in Table 1, 97.5% of the responding nurses were female and 2.0% were male. Among physicians, 42.1% were female and 57.9% were male. The majority of respondents were between 30 and 59 years of age. A significantly greater proportion of nurses worked in public health and obstetrics/gynaecology/newborn while fewer worked in family medicine. The proportion of physician respondents by specialty was very close to that found in Scott's Directory suggesting that non-response was similar in all four specialty groups. Just over 60% of all respondents had not received any formal training regarding woman abuse. Almost 50% of respondents indicated that they, a friend, or a relative had had experience with abuse, more than a third of respondents had called the CAS after an abuse disclosure and almost 18% had called the police after a disclosure.

**Table 1:** Demographic Characteristics of Study Sample, Percent with Characteristic

	**Full Study Sample (931)**	Nurses (597)*	Physicians (328)*
**SEX^**			
Female	77.6% (722)	97.5% (582)	42.1% (138)
Male	21.8% (203)	2.0% (12)	57.9% (190)
Missing	0.6% (6)	---	0
**AGE^(years)**			
20–29	6.9% (64)	9.4% (56)	2.1% (7)
30–39	24.9% (232)	24.1% (144)	26.8% (88)
40–49	33.1% (308)	32.0% (191)	35.7% (117)
50–59	28.8% (268)	28.6% (171)	29.0% (95)
60+	5.6% (52)	5.2% (31)	6.4% (21)
Missing	0.8% (7)	---	0
**CURRENT AREA OF PRACTICE^**			
Family Medicine	32.2% (300)	9.2% (55)	74.4% (244)
Emergency Medicine	21.2% (197)	27.1% (162)	10.4% (34)
Public Health	17.8% (166)	27.1% (162)	---
OB/gyn/newborn	22.6% (210)	30.0% (179)	9.1% (30)
Retired + other^+^	4.0% (37)	5.4% (32)	1.5% (5)
Missing	2.3% (21)	1.2% (7)	4% (13)
**ANY DISCLOSURES^**			
Never	23.1% (215)	31.2% (186)	7.9% (26)
None this year	10.3% (96)	11.9% (71)	7.6% (25)
Less than 20 this year	63.1% (587)	53.8% (321)	80.2% (263)
20 or more this year	2.5% (23)	1.8% (11)	3.7% (12)
Missing	1.1% (10)	1.8% (8)	---
**FORMAL IPV TRAINING**			
No	61.5% (573)	62.0% (370)	60.7% (199)
Yes	36.7% (342)	36.3% (217)	37.5% (123)
Missing	1.7% (16)	1.7% (10)	1.8% (6)
**RESPONDENT, FRIEND, OR RELATIVE EXPERIENCE^**			
No	49.7% (463)	45.4% (271)	57.3% (188)
Yes	48.4% (451)	52.9% (316)	40.9% (134)
Missing	1.8% (17)	1.7% (10)	1.8% (6)
**CALLED CAS^**			
No	61.5% (573)	66.8% (399)	52.4% (172)
Yes	36.3% (338)	31.0% (185)	46.0% (151)
Missing	2.1% (20)	2.2% (13)	1.5% (5)
**CALLED POLICE**			
No	81.2% (756)	81.6% (487)	80.5% (264)
Yes	17.6% (164)	17.4% (104)	18.3% (60)
Missing	1.2% (11)	1.0% (6)	---

* 6 did not indicate if they were a physician or a nurse; the number in the parenthesis is the sample size; ---: proportion suppressed, based on fewer than 5 observations; OB/gyn/newborn: Obstetrics/gynaecology/care of newborns; IPV: intimate partner violence;^+ ^: includes those who, at the time of the survey, worked in paediatrics, psychiatry, cardiac care, oncology, critical care and other areas; CAS: Children's Aid Society; ^: Z-tests indicate a statistically significant difference between Nurses and Physicians, p < 0.05

Several discipline-specific differences were noted. A significantly greater proportion of nurses compared to physicians had never heard a disclosure (31.2% of nurses vs. 7.9% of physicians, Z-test: 7.98, p < 0.001). A significantly higher proportion of nurses indicated that they, a friend, or relative had had experience with abuse (52.9% vs. 40.9%, Z-test: 3.42, p < 0.001), while more physicians had called the CAS (46.0% vs. 31.0%, Z-test: 4.47, P < 0.001).

### Reported behaviors

Factors associated with routine discussions of IPV were examined by profession, and discipline-specific differences were observed (Table 2). Physicians were significantly more likely to initiate the topic than were nurses (42.1% vs. 31.8%, Z-test: 3.08, p < 0.003), especially among clinicians working in the emergency department (52.9% vs. 24.7%, Z-test: 3.07, P < 0.003) and those who had worked in their current area of practice for less than 10 years (46.6% vs. 29.5%, Z-test: 3.27, P < 0.002). Within profession differences were also noted. Among nurses, those in public health were significantly more likely to initiate the topic than those working in other areas (Pearson Chi-square: 35.77, 5 df, p < 0.001).

**Table 2:** Reported Behaviors: Percent who agreed with "I routinely initiate the topic of woman abuse."

	**Full Study Sample**	**Nurses**	**Physicians**
**PROFESSION**			
Nurse	31.8% (190)		
Physician	42.1% (138)		
Missing	---		
**SEX**			
Female*	34.2% (247)	32.0% (186)	44.2% (61)
Male	39.4% (80)	---	40.5% (77)
Missing	---	---	0
**AGE **(years)			
20–29*	28.1% (18)	23.2% (13)	71.4% (5)
30–39*	32.8% (76)	26.4% (38)	43.2% (38)
40–49	34.1% (105)	29.8% (57)	41.0% (48)
50–59	40.7% (109)	39.2% (67)	44.2% (42)
60+	36.5% (19)	45.2% (14)	23.8% (5)
Missing	---	---	0
**CURRENT AREA OF PRACTICE**			
Family Medicine*	36.7% (110)	23.6% (13)	39.8% (97)
Emergency Medicine*	29.4% (58)	24.7% (40)	52.9% (18)
Public Health	49.4% (82)	49.4% (80)	---
OB/gyn/newborn	27.1% (57)	25.1% (45)	40.0% (12)
Retired + other	29.7% (11)	25.0% (8)	---
Missing	52.4% (11)	---	53.8% (7)
**YEARS IN CURRENT AREA OF PRACTICE**			
Less than 10 years*	35.0% (143)	29.5% (80)	46.6% (62)
10 years or more	36.0% (183)	34.0% (108)	39.7% (75)
Missing	---	---	---

The table shows the percent that endorsed both "agree" and "strongly agree"; the number in the parenthesis is the sample size; ---: proportion suppressed, based on fewer than 5 observations; OB/gyn/newborn: Obstetrics/gynaecology/care of newborns ;*: Z test indicates a statistically significant difference between Nurses and Physicians, p < 0.05

### Responses to individual items

Item non-response was very low, with missing values ranging from 0.6% to 2.9%. For many items there was little variability in response. In fact, more than 50% of all survey respondents selected one response option for all but three of the 43 questions ("I feel prepared asking about abuse of women who do not appear to me to be at risk of having been or being abused."; "I would not offer any assistance since there is no effective treatment for woman abuse."; and "I have opportunities for consultations regarding how to deal with situations such as Carol's.").

### Key constructs

Two study investigators initially identified eight constructs (preparedness, self-confidence, professional supports, abuse inquiry, practitioner consequences of asking, comfort following disclosure, practitioner lack of control, and practice pressures). However, there was disagreement as to the allocation of two of the 43 items ("It is an expectation to inquire about woman abuse." and "I would give her written information about woman abuse and/or available resources, but would not talk about her situation."). One investigator was unable to assign these two items to a scale, while the other did assign these items to specific scales.

Item and PCA analyses were also consistent with eight constructs (Table 3). Higher scores reflected greater self-reported preparedness, self-confidence, feelings of professional support, comfort with abuse inquiry, and comfort with discussions following a disclosure, as well as decreased concern about the consequences of abuse inquiry and decreased feelings of no control and system pressures.

**Table 3:** Item Analysis for Each Study Scale

	**Item Mean (SD)**	**Item-Total^#^**	**Alpha if Deleted**
**Preparedness **(n = 864)			
1. I would like to talk about the issue of abuse but don't know what to say^	2.93 (0.72)	0.62	0.86
2. I would be hesitant to ask about WA because I have little or no experience in dealing with this situation^	3.01 (0.68)	0.65	0.86
3. I feel prepared asking about abuse of women who appear to me to be at risk of having been or being abused*	2.91 (0.70)	0.67	0.85
4. I feel prepared asking about abuse of women who do not appear to me to be at risk of having been or being abused*	2.50 (0.71)	0.58	0.87
5. I feel ready to respond to a woman who says "no" to my question about abuse*	2.75 (0.62)	0.63	0.86
6. I feel ready to respond to a women who says "yes" to my question about abuse*	2.94 (0.61)	0.71	0.85
7. I feel prepared sharing information on woman abuse to clients who respond "no"*	2.65 (0.63)	0.54	0.87
8. I am hesitant to ask about woman WA because I have not been appropriately trained^	2.77 (0.74)	0.69	0.85
**Self-confidence **(n = 878)			
1. I am confident with my ability to address the issue of WA*	2.76 (0.71)	0.58	0.76
2. I feel that I am able to support this woman while she gets the right help*	2.92 (0.62)	0.53	0.77
3. I would feel confident if I were required to ask women about abuse*	2.83 (0.72)	0.53	0.77
4. I feel that I am a competent helper whether or not the woman and her situation change at this time*	2.88 (0.56)	0.60	0.76
5. I feel comfortable supporting the woman during the interview even though she may not be ready to deal with this problem in the same way I would want her to*	3.10 (0.55)	0.54	0.76
6. I feel comfortable discussing these practice situations with colleagues to help me deal effectively with WA*	3.10 (0.55)	0.37	0.79
7. I feel comfortable helping this woman access resources to help deal with the abuse*	2.98 (0.67)	0.53	0.77
**Practitioner lack of control **(n = 898)			
1. Since this is a private family matter, I should not interfere^	3.52 (0.56)	0.53	0.76
2. There isn't anything I can do unless she asks for help^	3.21 (0.60)	0.51	0.76
3. I would not ask her about WA because I don't think she is ready to tell me^	3.18 (0.57)	0.60	0.75
4. I feel that I am not able to help women who are abused^	3.21 (0.62)	0.48	0.77
5. I am reluctant to intervene in case I make matters worse^	3.15 (0.62)	0.61	0.74
6. I would not offer any assistance since there is no effective treatment for WA^	2.98 (0.60)	0.46	0.77
7. I would give her written information about WA and/or available resources, but would not talk about her situation^	3.48 (0.55)	0.42	0.78
**Comfort following disclosure **(n = 910)			
1. I feel I am able to listen to women's stories as they disclose the abuse they have experienced*	3.26 (0.54)	0.63	NA
2. I am able to continue the discussion after a disclosure to assess the needs of the client*	3.10 (0.59)	0.63	NA
**Professional supports **(n = 883)			
1. I feel comfortable discussing these practice situations with colleagues to help them deal effectively with woman abuse*	2.74 (0.71)	0.44	0.73
2. I have enough supports from colleagues, mentors, supervisors, etc. to help me feel comfortable in asking about woman abuse and in dealing with the responses*	2.77 (0.74)	0.51	0.69
3. I participate with my practice colleagues in planning and evaluating methods to develop or improve program delivery regarding WA*	2.20 (0.74)	0.56	0.66
4. I have opportunities for consultations regarding how to deal with situations such as Carol's*	2.48 (0.79)	0.61	0.62
**Practice pressures **(n = 887)			
1. I may forget to ask her about WA^	2.97 (0.70)	0.41	0.72
2. I just don't have time today to address this possible abuse issue^	3.12 (0.65)	0.53	0.67
3. I am reluctant to ask about WA because there are not sufficient community resources to provide assistance^	3.02 (0.64)	0.52	0.67
4. I am hesitant to ask about WA because I might have to call the CAS or the police^	3.15 (0.64)	0.51	0.68
5. I feel frustrated because I don't have the time to talk about abuse^	2.65 (0.73)	0.50	0.68
**Abuse inquiry **(n = 875)			
1. I routinely initiate the topic of WA*	2.33 (0.72)	0.50	0.65
2. I would ask her directly if her husband has ever hit her*	2.98 (0.74)	0.47	0.66
3. I won't put her on the spot by initiating the topic of abuse^	2.96 (0.63)	0.46	0.67
4. I am hesitant to ask about WA in case the woman stops seeing me^	3.13 (0.55)	0.41	0.68
5. I am hesitant to ask some clients about WA because to them it is culturally acceptable^	3.04 (0.57)	0.32	0.70
6. I would introduce WA by stating that abuse frequently occurs and that often women are hesitant to talk about it*	2.92 (0.67)	0.41	0.68
7. It is an expectation to inquire about WA*	2.79 (0.69)	0.37	0.69
**Practitioner consequences of asking **(n = 896)			
1. I worry about my own safety when inquiring about WA^	3.06 (0.66)	0.40	0.49
2. I think about possible legal consequences when asking about WA^	2.81 (0.70)	0.39	0.50
3. I am hesitant to ask about WA because I also treat/deal with other family members^	3.06 (0.58)	0.41	0.48

item-total^#^: Item-total correlation; WA: Woman abuse, CAS: Children's Aid Society; NA: Not applicable

Items scoring ^: strongly agree (1), agree (2), disagree (3), strongly disagree (4)

*: strongly agree (4), agree (3), disagree (2), strongly disagree (1)

Based on an examination of item-item and item-total correlations, one of the items ("I feel comfortable discussing these practice situations with colleagues to help me deal effectively with woman abuse.") originally allocated to the construct professional supports, was reassigned to the construct self-confidence. Item analyses also suggested that the item "It is an expectation to inquire about woman abuse." was associated with the items that were part of the construct abuse inquiry and that "I would give her written information about woman abuse and/or available resources, but would not talk about her situation." was associated with items that were part of the construct practitioner lack of control. Thus, three survey items were assigned to scales based solely on statistical relationships.

Construct-specific item analyses showed that in all cases, the scale alpha would have either stayed the same or decreased if the item had been deleted and all item-total correlations were greater than 0.30 (Table 3) suggesting that each scale measured a single construct.

As noted in Table 4, seven of the eight scales demonstrated "acceptable" internal consistency (alpha greater than 0.7) required for group comparisons [32]. It is not surprising that the scale with the lowest internal consistency was practitioner consequences of asking (alpha 0.59) as this scale included only three items and alpha decreases with decreasing numbers of scale items. For each of the eight constructs, factor loadings associated with each item were greater than 0.45. Further, the percent of the total variance explained by each construct varied from 36.6% (abuse inquiry, seven items) to 81.3% (comfort following disclosure, two items). While in theory scale scores could range from one to four, this was only the case for two of the eight scales (professional supports and comfort following disclosure). The scale with the narrowest range was abuse inquiry (observed range, two to four).

**Table 4:** Constructs Measured in Survey Instrument

**Construct**	**# items**	Alpha*	**% Var. Explained**	**Factor Loadings**	**Mean (SD)**	**Score Range**
Preparedness	8	0.87	53.6%	0.78-.064	2.80 (0.50)	1.37–4.00
Self-confidence	7	0.79	45.2%	0.74-0.50	2.94 (0.42)	1.57–4.00
Practitioner lack of control	7	0.79	44.4%	0.75-0.57	3.24 (0.39)	1.57–4.00
Comfort following disclosure	2	0.77	81.3%	0.90	3.10 (0.51)	1.00–4.00
Professional supports	4	0.74	56.0%	0.82-0.66	2.55 (0.56)	1.00–4.00
Practice pressures	5	0.73	48.4%	0.73-0.60	2.98 (0.47)	1.60–4.00
Abuse inquiry	7	0.71	36.6%	0.68-0.49	2.88 (0.40)	2.00–4.00
Practitioner consequences of inquiry	3	0.59	55.0%	0.75-0.74	2.98 (0.48)	1.33–4.00

Alpha*: Chronbach's Alpha; % Var. Explained: percent of total item variance explained by factor; SD: standard deviation

### Construct validity: associations with experience and education

Scale scores increased with experience with abuse disclosure (Table 5). For all constructs, the lowest scale scores were associated with never having heard a disclosure and the highest scores were associated with 20 or more disclosures in the last year. The largest scale score increases were observed for preparedness and professional supports. The smallest increase was associated with practitioner consequences of asking.

**Table 5:** Mean Score by Construct and by Annual Number of Abuse Disclosures

	**Never**	**None this year**	**1–19**	**20+**	**Missing**
Preparedness	2.45 (0.46)	2.88 (0.48)^	2.90 (0.44)^	3.34 (0.44)^* ^#^	3.03 (0.60)
Self-confidence	2.70 (0.37)	2.97 (0.43)^	2.99 (0.39)^	3.42 (0.44)^* ^#^	3.00 (0.63)
Practitioner lack of control	3.07 (0.39)	3.28 (0.35)^	3.29 (0.38)^	3.54 (0.36)^	3.26 (0.40)
Comfort following disclosure	2.88 (0.51)	3.14 (0.44)^	3.16 (0.49)^	3.46 (0.47)^	3.33 (0.56)
Professional supports	2.36 (0.48)	2.56 (0.54)	2.59 (0.56)^	3.19 (0.58)^* ^#^	2.46 (0.57)
Practice pressures	2.88 (0.39)	3.03 (0.42)	3.00 (0.49)^	3.34 (0.55)^	2.67 (0.42)
Abuse inquiry	2.62 (0.30)	2.88 (0.37)^	2.96 (0.38)^	3.26 (0.38)^*	2.98 (0.43)
Practitioner consequences of asking	2.86 (0.46)	2.98 (0.44)	3.01 (0.49)^	3.18 (0.45)	2.63 (0.61)

Number in parenthesis is the standard deviation associated with the mean; one-way ANOVA, Dunnett's t-test used to test significance of post hoc tests; ^: significantly higher than "never"; *significantly higher than "none this year"; #: significantly higher than "1–19 this year"; all cases p < 0.01

Mean scale scores were also examined by training and by experience (Table 6). Although the scale score associated with "training, no experience" was higher than "no training, experience" for all but one construct (professional supports), for all eight constructs the difference between these two categories was not statistically significant. When compared to "no training, no experience", two construct scale scores increased significantly with just training (preparedness and abuse inquiry), while five construct scale scores increased with just experience (preparedness, self-confidence, abuse inquiry, comfort following disclosure, and practitioner lack of control). In all cases, the scores associated with both training and experience were significantly higher than the scores associated with "no training, no experience". The largest scale score increase was observed for preparedness and the smallest for both practitioner consequences of asking and practice pressures.

**Table 6:** Mean Score by Construct and by Training and Experience

	**No training, No experience**	**Training, No experience**	**No training, experience**	**Training, experience**	**Missing**
Preparedness	2.39 (0.45)	2.67 (0.42)^	2.77 (0.43)^	3.10 (0.43)^*^#^	2.99 (0.46)^
Self-confidence	2.67 (0.38)	2.84 (0.31)	2.90 (0.37)^	3.15 (0.41)^*^#^	2.93 (0.49)
Practitioner lack of control	3.03 (0.39)	3.19 (0.36)	3.22 (0.36)^	3.41 (0.37)^*^#^	3.31 (0.38)
Comfort following disclosure	2.84 (0.53)	3.00 (0.43)	3.09 (0.46)^	3.28 (0.51)^*^#^	3.11 (0.44)
Professional supports	2.31 (0.48)	2.56 (0.47)	2.44 (0.51)	2.82 (0.56)^^#^	2.66 (0.52)
Practice pressures	2.88 (0.38)	2.89 (0.41)	2.94 (0.45)	3.11 (0.50)^^#^	2.87 (0.50)
Abuse inquiry	2.58 (0.29)	2.77 (0.30)^	2.86 (0.35)^	3.08 (0.40)^*^#^	2.93 (0.36)
Practitioner consequences of asking	2.85 (0.48)	2.91 (0.37)	2.97 (0.47)	3.08 (0.49)^	2.80 (0.56)

Number in parenthesis is the standard deviation associated with the mean; one-way ANOVA, Dunnett's t-test used to test significance of post hoc tests; ^: significantly higher than "no training no experience"; *: significantly higher than "training, no experience"; #: significantly higher than "no training, experience"; all cases p < 0.01

### Modelling the relationship between preparedness and training and experience

As previously discussed, mean preparedness scores increased significantly with training or experience or both training and experience (no training, no experience: 2.39; training, no experience: 2.67; no training, experience: 2.77; training and experience: 3.10; missing: 2.99; F: 71.88, 4 df, p < 0.001). Those with training and experience had significantly higher scores than those with training and no experience and those with no training but with experience. However, the scores associated with "training, no experience" and "no training, experience" were not significantly different. These results suggest a possible interaction between training and experience. Bivariate analyses also suggested that the categories "none this year" and "1 to 19 disclosures" could be collapsed and a histogram of preparedness scores showed that the distribution was fairly normal (kurotis: 0.158 (SE 0.164) and skewness: 0.115 (SE 0.082)). Thus, two linear regression models were generated, one for those who had never received any formal training and another for those who had received formal training.

Among those who did not have any formal training, exploratory forward, backward, and stepwise models suggested four independent variables (profession, years in current practice location, level of professional experience, and respondent, friend, or relative experience). Nurses had a significantly lower preparedness score than physicians (unstandardized regression coefficient (β): -0.14, t-test: -3.48, p < 0.01). Further, preparedness scores increased as the level of experience increased (when compared to no disclosures, less than 20 disclosures that year increased preparedness scores by 0.30 (t-test: 6.93, p < 0.001) while 20 or more disclosures increased preparedness by 0.50 (t-test: 2.78, p < 0.01)). Respondent, friend, or relative experience with abuse increased preparedness scores by 0.11 (t-test: 2.78, p < 0.01). And, preparedness scores increased by 0.08 (t-test: 2.06, p < 0.05) among those who had 10 or more years of experience in their current practice setting when compared to those who had less than 10 years experience. Other experience with abuse disclosure (calling police or CAS) did not seem to be associated with a change in preparedness scores in this group. The adjusted R^2 ^for this model was 16.7.

Among those who did receive formal training, exploratory forward, backward, and stepwise models suggested three independent variables (level of professional experience, respondent, friend, or relative experience, and having called the CAS). For this group, profession had no impact on preparedness scores. Again, preparedness scores increased with level of experience (when compared to no disclosures, less than 20 disclosures that year increased preparedness scores by 0.24 (t-test: 3.30, p < 0.01) while 20 or more disclosures increased preparedness by 0.64 (t-test: 4.74, p < 0.01)) and respondent, friend, or relative experience (β: 0.12, t-test: 2.45, p < 0.05). Further, if the respondent had called the CAS in the past, their preparedness score significantly increased (β = 0.20, t-test: 4.10, p < 0.001). As indicated earlier, the study question did not stipulate if calls to the CAS were related to clinical or to personal experience. The adjusted R^2 ^for this model was 16.2.

## Discussion

The present study adds new insight into HCPs' perceptions about barriers and facilitators to routine inquiry about woman abuse. We found that both training and professional experience are associated with increased feelings of preparedness and self-confidence, promotion of professional networks, help with comfort initiating discussions of IPV, decreased anxiety about negative consequences of asking, increased comfort with discussions following abuse disclosure, practitioners feeling more in control, and decreased effects of practice pressures.

Almost 50% of respondents indicated that they, a friend, or a relative had had experience with abuse, highlighting the prevalence of this issue. However, over 60% of physicians and nurses responding to the present survey reported not having received specific training in this area, a finding consistent with a recent Ontario-wide survey of colleges, universities and professional organizations regarding IPV-specific educational opportunities provided to health care providers in undergraduate, post-graduate, and continuing education [33]. Overall, 83% of colleges and university undergraduate nursing programs, and 43% of undergraduate medical programs offered at least some (mostly minimal) exposure to IPV-related content in the curriculum (these figures were far lower at the post-graduate/continuing education levels). Thus it is not surprising that the majority of practicing physicians and nurses responding to this survey indicated lack of adequate formal training.

Among those with no formal IPV education in the present survey, professional differences were noted in preparedness to address IPV, with nurses feeling less prepared than physicians, an interesting finding with no clear explanation. These professional differences in preparedness disappear when training is present.

While formal education is important [34], professional experience with abuse disclosures is perhaps the key factor influencing how prepared clinicians feel to address IPV. There was also some indication that respondent, friend, or relative experience with IPV is associated with preparedness, but not to the same extent as having talked about IPV with abused women.

Our findings enhance and extend those of previous studies that have examined clinician attitudes and practices specific to IPV identification and intervention. The key factors affecting readiness to identify and respond to IPV identified in the literature include: gaps in provider knowledge and lack of education regarding IPV; the perception of a lack of patient compliance (patient does not disclose); lack of effective interventions; and perceived system support, especially time. Other factors include provider self-efficacy, including feelings of powerlessness, and loss of control; safety concerns and fear of offending; affective barriers (e.g., lack of comfort, interest, and sympathy); poor interviewing or communication skills; providers' personal experience with abuse; and their age and years in practice [17,21-27,29,30,33-37].

Finally, a recent meta-synthesis of qualitative studies by Feder and colleagues [38] identified appropriate HCP training as a basic expectation that women have if they are going to be asked about abuse. Based on the synthesis and interpretation of data from 25 studies that explore women's experiences of disclosure to HCPs, the authors conclude that, prior to inquiry about abuse, women require that the HCP "(h)ave a full understanding of the issue of domestic violence, including knowledge of community services and appropriate referrals" [[38], p. 36]. However these authors point out that the context and consequences of routine inquiry, including how and how often to ask about abuse, depend on a number of factors, including the woman's readiness to address the violence or leave the relationship, her perceived safety, and her concerns for her children, and ensuring that the inquiry is appropriate to the context of the clinical encounter [38].

There were several potential limitations to the present study. Only 32.8% of physicians contacted returned a completed questionnaire. While this response rate is consistent with random surveys of physicians in general [28], if only those with strong opinions about the topic responded, the results for this group may be biased. Also, only one physician from public health responded, making it impossible to generalize these findings to public health physicians. However, the sample sizes in other profession-based practice areas, such as family physicians and nurses in public health, emergency, and obstetrics/gynecology were larger, making the findings in these groups more robust.

This study was a descriptive cross-sectional study, and while we can provide data on the strength of association between specific factors and HCPs' preparedness and behaviours regarding routine inquiry for IPV, we cannot comment on whether these factors predict this behaviour. The questions also have specific limitations. The selection of items, while based on the literature and consultation with IPV experts, was not theory-driven. Therefore, we may have missed some key concerns or concepts.

The open-ended questions included in the survey instrument yielded responses from 766 individuals (526 nurses, 236 physicians, and four did not indicate their profession). Respondents used these questions to share their practice experiences and personal stories, offer their opinions and to ask questions about screening. This suggests that attitudes and behaviours regarding screening are very complex, and perhaps so context specific that using a questionnaire to identify barriers and facilitators may be overly simplistic and superficial.

Future research in this area should include studies with representative samples that examine the variations in practice within and between the different types of health care providers and sub-specialties identified as key first responders to abused women.

## Conclusion

In summary, the present study adds to our understanding of the barriers and facilitators identified by health care providers to appropriately identifying female patients who may be exposed to violence. Our findings highlight the significant impact that IPV-specific training and professional experience have on clinicians' self-reported attitudes and practices regarding inquiry about IPV. Certain expectations often cited in the IPV literature, and among leaders advocating an active and consistent response by health care professionals, may not match the realities of the health care provider's professional preparation and practice context. Finding a better match between these expectations and clinicians' realities will provide the best context for an appropriate health care response for abused women, and educational efforts that integrate established and emerging evidence about how best to recognize, ask about and respond to IPV in health care settings are an urgent priority.

## Competing interests

The author(s) declare that they have no competing interests.

## Authors' contributions

IG and CB designed and conducted the survey with critical input from LT and HLM. IG analysed the results and prepared the initial draft of the Background, Methods and Results sections. CNW prepared the initial draft of the Introduction and Discussion sections. CB, LT and HLM critically reviewed and approved the final manuscript.

## Pre-publication history

The pre-publication history for this paper can be accessed here:



## Supplementary Material

Additional File 1 Study questionnaire instructions and practice scenario. Click here for file
