# Supplementary material for: Factors influencing identification of and response to intimate partner violence: a survey of physicians and nurses
Source: BMC Public Health. 2007 Jan 24;7:12. doi: 10.1186/1471-2458-7-12 (PMC1796870; doi:10.1186/1471-2458-7-12)
Supplement: Additional File 1 — Study questionnaire instructions and practice scenario. [file 1471-2458-7-12-S1.doc]

# Additional files

### Study questionnaire instructions and practice scenario

# Instructions

We are providing a scenario to reflect a real life situation. Please respond to the following statements in a way that would reflect either your current practice or your hypothetical response to the scenario. Do not mark the questionnaire in any way that would identify you by name or address.

# Practice scenario

Carol is pregnant and has a toddler, Jennifer. Carol appears quiet, withdrawn and she says that she is very tired. When you ask Carol what help she has in caring for the toddler and the house, she says that she does it all herself. She comments that her husband believes that it is her job to keep the house clean and to keep the toddler from bothering him when he is home. She adds that he criticizes her frequently. She does not go out of the house unless her husband drives her. However, her greatest concern is how to keep the toddler quiet, since her husband gets extremely angry with her every time the toddler "acts up". Carol sounds agitated when she talks about this. Although her mother, sister and two old friends are in the city, Carol doesn't see them much any more and she does not know her neighbours.

When working with Carol or other women in your practice setting, please indicate the extent to which you would agree/disagree with the statements below.
